# Resveratrol, a Natural Antioxidant, Has a Protective Effect on Liver Injury Induced by Inorganic Arsenic Exposure

**DOI:** 10.1155/2014/617202

**Published:** 2014-07-24

**Authors:** Zhigang Zhang, Li Gao, Yanyan Cheng, Jing Jiang, Yan Chen, Huijie Jiang, Hongxiang Yu, Anshan Shan, Baojing Cheng

**Affiliations:** ^1^College of Veterinary Medicine, Northeast Agricultural University, Harbin 150030, China; ^2^Laboratory of Molecular Nutrition and Immunity, The Institute of Animal Nutrition, Northeast Agricultural University, Harbin 150030, China

## Abstract

Resveratrol (Rev) can ameliorate cytotoxic chemotherapy-induced toxicity and oxidative stress. Arsenic trioxide (As_2_O_3_) is a known cytotoxic environmental toxicant and a potent chemotherapeutic agent. However, the mechanisms by which resveratrol protects the liver against the cytotoxic effects of As_2_O_3_ are not known. Therefore, in the present study we investigated the mechanisms involved in the action of resveratrol using a cat model in which hepatotoxicity was induced by means of As_2_O_3_ treatment. We found that pretreatment with resveratrol, administered using a clinically comparable dose regimen, reversed changes in As_2_O_3_-induced morphological and liver parameters and resulted in a significant improvement in hepatic function. Resveratrol treatment also improved the activities of antioxidant enzymes and attenuated As_2_O_3_-induced increases in reactive oxygen species and malondialdehyde production. In addition, resveratrol attenuated the As_2_O_3_-induced reduction in the ratio of reduced glutathione to oxidized glutathione and the retention of arsenic in liver tissue. These findings provide a better understanding of the mechanisms whereby resveratrol modulates As_2_O_3_-induced changes in liver function and tissue morphology. They also provide a stronger rationale for the clinical utilization of resveratrol for the reduction of As_2_O_3_-induced hepatotoxicity.

## 1. Introduction

Arsenic trioxide (As_2_O_3_) is a known cytotoxic environmental toxicant and is used as a potent chemotherapeutic agent for acute promyelocytic leukemia (APL) and other cancers [1]. Retrospective case studies have shown a significant association between toxicity and the degree of retention of toxic compounds in tissues [2, 3]. Arsenic interferes with a number of organ and body functions such as the central nervous system [4], liver, and kidney [5, 6]. Liver as a major organ is responsible for the metabolism of drugs and is also the primary target for many toxic chemicals, with evidence of the highest concentration of total arsenic retention following acute arsenic toxicity [7]. In experimental animals, arsenicals induce liver injuries that cause alterations in the biochemical indices of hepatic functions, as well as changes in the morphology and apoptosis of liver cells [8, 9].

Both the toxic and therapeutic effects of arsenic are partially mediated by redox-sensitive enzymes and proteins. Ghosh et al. reported that the free radical mediated theory is one of the theories proposed to explain As_2_O_3_-induced injury [9]. Reactive oxygen species (ROS) are involved in normal cellular metabolism and signal transduction. However, they can cause damage at higher concentrations and induce several human pathologies, including liver cirrhosis and fibrosis [10]. Therefore, synthetic scavengers of ROS and antioxidants are to reduce arsenic-induced toxic effects.

Resveratrol (Rev) (trans-3,4′,5-trihydroxystilbene) is isolated as a natural phytoalexin contained in wine and has several beneficial effects on health. Over the years, this molecule has received considerable attention for its anti-inflammatory [11], antiviral [12], and antioxidant properties [13–15], as well as its ability to increase lifespan in lower organisms and improve general health in mammals [16]. The main target organs of resveratrol are the liver and kidney, as has been demonstrated in pharmacokinetic and pharmacodynamic studies [17]. Resveratrol exerts hepatoprotective activity against ethanol- and thioacetamide-induced acute liver damage in rats [18]. Systemic administration of resveratrol has since been shown to inhibit the initiation and growth of tumors in a wide variety of rodent cancer types [19]. Accumulating evidence also indicates that resveratrol has the capacity to exert sensitization effects when it is used in combination with cytotoxic drugs in drug-resistant tumor cells [20, 21].

Although the beneficial properties of resveratrol have been well defined, the mechanisms by which resveratrol protects the liver against the cytotoxic effects of As_2_O_3_ are not known. Therefore, the present study was undertaken to evaluate whether or not resveratrol can ameliorate the toxic effects of As_2_O_3_ in terms of recovery in biochemical variables indicative of oxidative stress, tissue injuries, and reduction in liver arsenic burden. These findings could provide a better understanding of the mechanisms of action of resveratrol in modulating As_2_O_3_-induced hepatic dysfunction and provide a rationale for further clinical study of resveratrol used in combination with As_2_O_3_ during chemotherapy or as a protective agent after As_2_O_3_ exposure.

## 2. Materials and Methods

### 2.1. Materials

As_2_O_3_ parenteral solution (10 mg/mL) was obtained from Harbin Yida Pharmaceutical Company Ltd. (Harbin, China). Resveratrol was provided by Sigma-Aldrich (St. Louis, MO, USA). Kits used for the detection of superoxide dismutase (SOD), catalase (CAT), glutathione peroxidase (GPX), malondialdehyde (MDA), glutathione (GSH), and glutathione disulfide (GSSG) were purchased from Jiancheng Bioengineering Institute (Nanjing, China). All other chemicals used were of the highest purity commercially available and purchased from Shanghai Biochemical and Beijing Chemical Co. Triple distilled water was used throughout the study to avoid metal contamination and for the preparation of reagents and buffers used for various biochemical assays.

### 2.2. Animals and Treatment

As the pharmacokinetic profiles of drugs in cats and humans are similar, cats are suitable for pharmacology and toxicology studies [22, 23]. The study was performed using healthy Chinese Dragon-Li cats (12 males and 12 females) maintained in air-conditioned rooms at 21 ± 4°C. The cats were housed in individual stainless steel cages (during experimental phases). Weights and ages ranged from 2.8–3.5 kg to 1.5–2.0 years, respectively.

All animals were randomly divided into four groups: control, As_2_O_3_-treated, As_2_O_3_ + resveratrol-treated, and resveratrol-treated. All of the treatments used in the study were administered* via* the foreleg vein on alternate days for 3 days (i.e., days 1, 3, and 5) with measurements made on the 6th day. Cats were treated as follows: in the control group they were injected with 10 mL/kg physiological saline (0.9%); in the As_2_O_3_ group they were treated with As_2_O_3_ (1 mL/kg); in the As_2_O_3_ + resveratrol (3 mL/kg) group they were given resveratrol (3 mL/kg) 1 h before As_2_O_3_ administration. The resveratrol “control” group received three doses of resveratrol alone (3 mL/kg).

The selection of a single dose of resveratrol was based on previous published reports, where a dose of 3 mL/kg was reported to be effective in preventing diverse biological and pharmacological properties [24].

Twenty-four hours after the last injection, all cats were killed by an overdose of pentobarbital sodium. Blood was collected and liver was rinsed in cold saline and used for evaluating various biochemical variables as well as arsenic concentrations. All study procedures were approved by Ethical Review Committees at the Northeast Agricultural University, China.

### 2.3. Preparation of Plasma and Liver Parameters

Some of the blood samples were collected into evacuated tubes containing heparinic solution as anticoagulant and then centrifuged at 3000 g for 10 min. The activities of alanine aminotransferase (ALT) and aspartate aminotransferase (AST) and the levels of total bilirubin and cholesterol were detected using a UniCel DxC800 Synchron chemistry system (Bekman, USA).

### 2.4. Collection of Liver Samples and Biochemical Determination

Liver tissues were rapidly excised and homogenized in phosphate-buffered saline pH 7.4 using an Ultrathurax T25 Homogenisator. After centrifugation at 10,000 g for 10 min at 4°C, the supernatant was used for biochemical determination. The MDA, GSH, and GSSG levels, hepatic proteins, and SOD, CAT, and GPX activities in tissue were determined according to the manufacturer's instructions. The GSH/GSSG ratio was calculated as follows: ratio = [(GSH) − 2(GSSG)]/(GSSG).

### 2.5. Measurement of ROS Level in Liver

The amount of ROS in liver was measured by the method of 2′,7′-dichlorofluorescein diacetate, which is converted into highly fluorescent dichlorofluorescein by cellular peroxides (including hydrogen peroxide). The assay was performed as described in the literature [25]. Fluorescence was determined at 488 nm excitation and 525 nm emission wavelength, using a fluorescence plate reader (Perkin-Elmer, LS-55, Buckinghamshire, UK).

### 2.6. Morphological Examination

Liver tissues from cats were fixed in 10% formaldehyde for overnight at 37°C. Tissues were cut into blocks of 3 mm thickness. Blocks were then embedded in paraffin. Sections (5 *μ*m thickness) were cut in the coronal plane and stained with hematoxylin and eosin. Morphological examination was conducted under a light microscope (BX-FM: Olympus Corp, Tokyo, Japan).

### 2.7. Determination of Total Arsenic in the Liver

A liver tissue sample of approximately 0.5 g was digested in a solution consisting of a mixture of HNO_3_-HCLO_4_ for 2 days at 130°C. After HNO_3_ was removed by evaporation, digested samples were diluted with deionized water. Arsenic concentrations were measured using atomic fluorescence spectrometry (AFS930, Beijing Jitian Instrument Company, Beijing, China) [26].

### 2.8. Statistical Analysis

Data represent the mean ± SEM. Statistical analyses were undertaken by one-way ANOVA and the Student's *t*-test. A two-tailed *P* < 0.05 was considered as being significant.

## 3. Results

**Figure 1:**
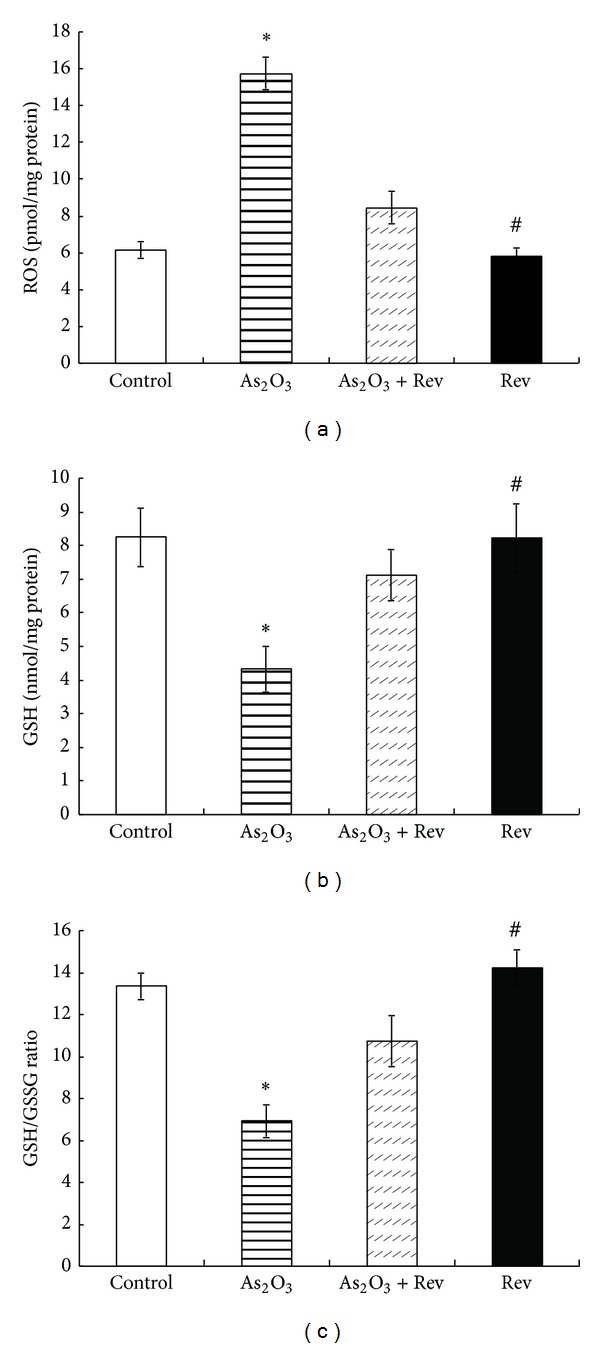
Arsenic-induced change in ROS, GSH, and GSH/GSSG ratio in liver and their response to pretreatment with resveratrol. Values are mean ± SEM; *P < 0.05 versus control group and ^#^ *P* < 0.05 versus As_2_O_3_-treated group.

The liver ROS level increased on arsenic exposure (Figure 1(a)). Pretreatment with resveratrol was beneficial in significantly reducing tissue ROS levels in the As_2_O_3_-treated group.

We analyzed the overall levels of GSH and GSSG in liver and calculated their ratio and found that As_2_O_3_ treatment significantly reduced the levels of GSH (Figure 1(b)) and the GSH/GSSG ratio (Figure 1(c)), whereas resveratrol treatment attenuated this reduction. Treatment with resveratrol alone did not cause any significant changes in the GSH/GSSG ratio relative to the control.

**Figure 2:**
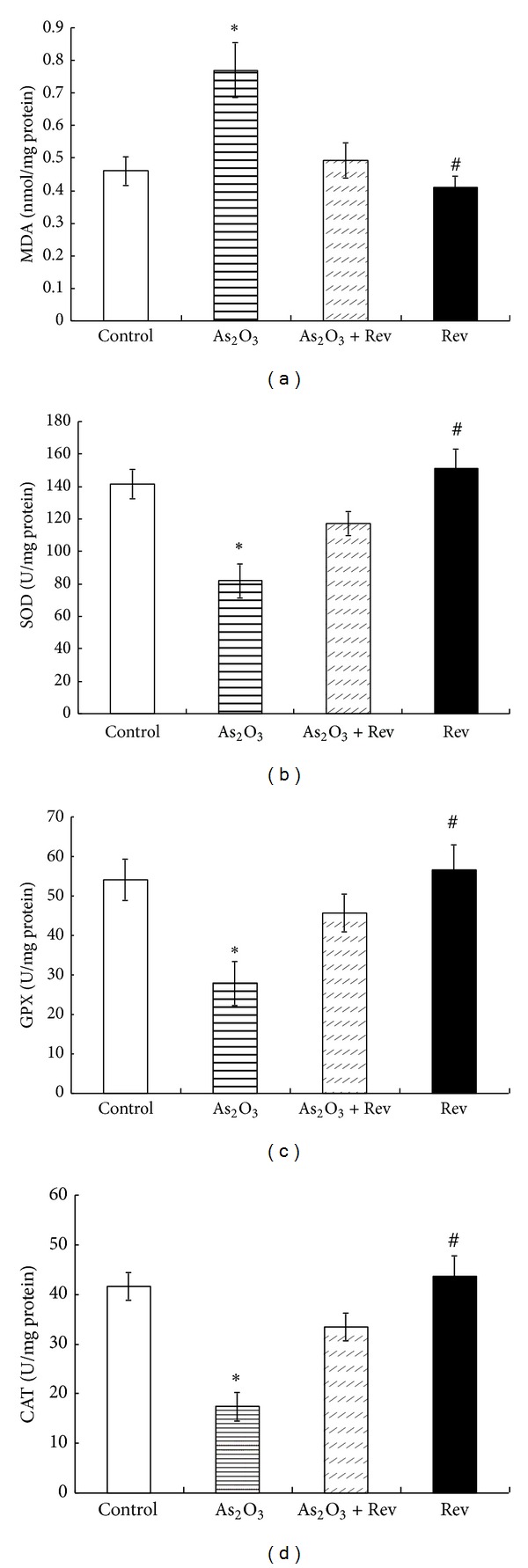
Arsenic-induced changes in SOD, GPX, CAT, and MDA in liver and their response to pretreatment with resveratrol. Values are mean ± SEM; *P < 0.05 versus control group and ^#^ *P* < 0.05 versus As_2_O_3_-treated group.

Figure 2(a) shows the effect of resveratrol on hepatic MDA levels. We found a significant increase in liver MDA after arsenic exposure. Pretreatment with resveratrol resulted in a pronounced decrease in MDA level relative to arsenic exposed animals, suggesting a beneficial role of the resveratrol on lipid peroxidation.

Figure 2 depicts the effect of pretreatment with resveratrol on the antioxidant enzyme systems SOD (Figure 2(b)), GPX (Figure 2(c)), and CAT (Figure 2(d)). There was a significant depletion of SOD, GPX, and CAT activity after arsenic exposure. Treatment with resveratrol prevented the depletion of SOD, GPX, and CAT activity.

Treatment with arsenic caused a significant increase in the total bilirubin, cholesterol, AST, and ALT levels, relative to the control (Table 1). However, treatment with resveratrol alone did not cause any significant changes in the activities of AST and ALT as compared with the control; pretreatment with resveratrol resulted in recovery in the above-mentioned biochemical variables.

**Figure 3:**
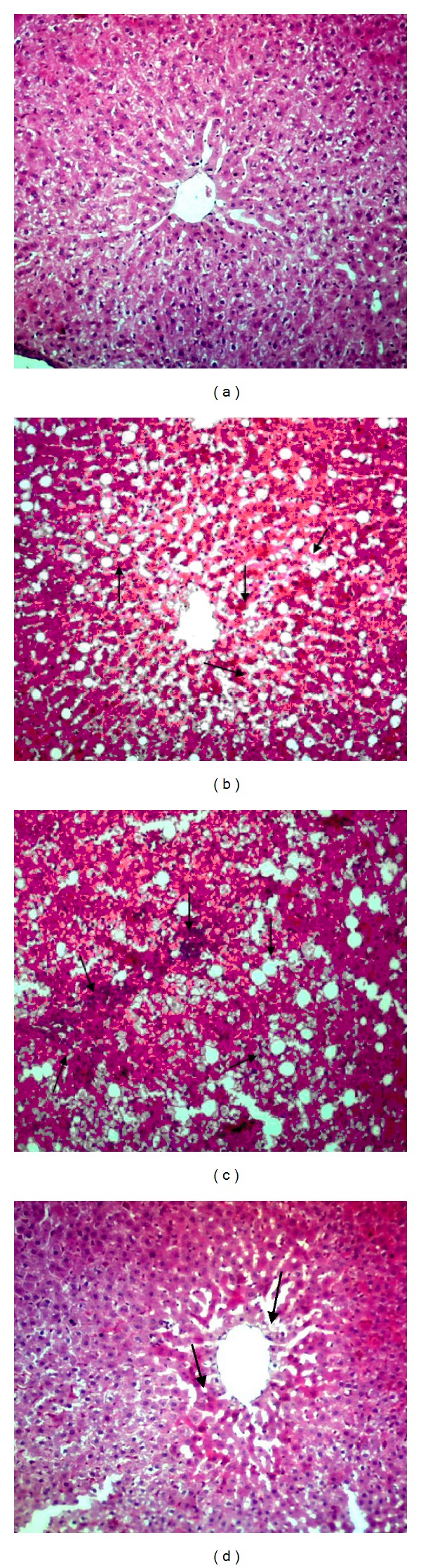
Liver histology in the control animals (a), As_2_O_3_ group (b) and (c), and As_2_O_3_ + resveratrol group (d) (H&E, ×100). Increased cytoplasmic vacuolization, focal necrosis, and inflammatory cell infiltration were noted in (b) and (c). (d) showed slight cytoplasmic vacuolization.

The biochemical alterations mentioned above could be correlated with the histological changes in the liver shown in Figure 3. Histopathology of liver in the control group revealed a normal structure with a regular arrangement of hepatocytes with clearly visible nuclei and a characteristic pattern of hexagonal lobules (Figure 3(a)). After As_2_O_3_ treatment the liver exhibited serious pathological alterations such as the presence of increased cytoplasmic vacuolization, focal necrosis, and inflammatory cell infiltration (Figures 3(b) and 3(c)). The livers of resveratrol-treated cats (Figure 3(d)) showed good protection against hepatocellular necrosis, with a regular arrangement of hepatocytes and a slight cytoplasmic vacuolization around the central vein; hepatocytes of animals treated only with resveratrol had a normal morphology (data not shown).

**Figure 4:**
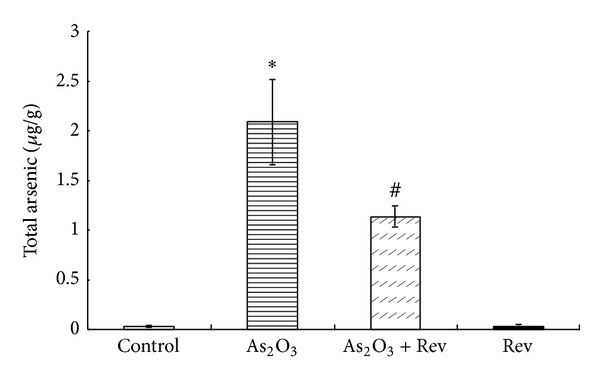
Total arsenic retention in cat livers. Data are mean ± SEM; *P < 0.01 versus control group and ^#^ *P* < 0.05 versus As_2_O_3_-treated group.

Figure 4 shows the effect of pretreatment with resveratrol on arsenic concentrations in liver. Exposure to arsenic resulted in a significant increase in the arsenic concentration of the liver relative to the control. Administration of resveratrol significantly reduced the liver arsenic concentration.

## 4. Discussion

The mechanisms by which As_2_O_3_ causes hepatic dysfunction are not yet completely understood, but inducting the production ROS in tissues is one of the reasons [27, 28]. The amount of ROS in cells is dependent on both the production of ROS by the mitochondrial electron transport chain and their removal by ROS-detoxifying enzymes. Recently, some researchers have evaluated the effects of arsenic intermediate metabolites (monomethylarsonous acid, MMA^III^, and trivalent dimethylarsinous acid, DMA^III^) on the induction of ROS. It has been clearly confirmed that the generation of ROS is specifically induced in the endoplasmic reticulum by exposure to DMA^III^, while previous studies have shown that MMA^III^ induces ROS generation specifically through inhibition of the activities of complexes II and IV in mitochondria [29, 30]. In accordance with previous study, it was observed in our research that ROS level was markedly increased after arsenic exposure [31]. However, the role of resveratrol in mediating hepatic dysfunction induced by As_2_O_3_ is not well understood. Our study demonstrated that the addition of resveratrol improved the protection of the liver against the cytotoxic effects of this agent, including hepatic function; this was suggested by liver parameters and liver histological changes. The protective effects of resveratrol against As_2_O_3_ may be attributable to its ability to modulate cellular redox. This suggestion is supported by the finding that resveratrol treatment persevered the activities of SOD, CAT, and GPX, inhibited As_2_O_3_-induced increases in ROS production, and attenuated As_2_O_3_-induced reduction in the ratio of GSH/GSSG in liver. MDA (the end product of lipid peroxidation) is known as an index that can be used to monitor the oxidative damage. However, in the present study pretreatment with resveratrol induced the marked decrease in MDA levels relative to the As_2_O_3_-treated group, which might be attributable to the above findings. These data related to the beneficial effects of resveratrol could be explained by the important role that it plays in improving the activity of mitochondrial complexes I, III, and IV [32], as well as imbalance in the levels of ROS and antioxidants [33, 34]. Also, it has been proposed that resveratrol indirectly activates PGC-1*α*, which can regulate mitochondrial energetics and induce the expression of many ROS-detoxifying enzymes including SOD, CAT, and GPX [35, 36]. As resveratrol can effectively protect liver by inhibiting As_2_O_3_-induced ROS production, it may decrease the risk of cancer patients developing secondary malignancies. Since chemotherapy can induce increased ROS production which causes genetic instability, then the risk of leukemia and cancer will be increased [37].

One of the most interesting observations in our study was the ability of resveratrol to reduce the concentration of arsenic in the target tissue, which may play a crucial role in its hepatoprotective effect. Protection against arsenic in human cells has been shown to be associated with GSH-dependent efflux from the cell [38]. Depleting GSH has been reported to increase sensitivity to arsenic and its retention in mammalian cells [39, 40]. To explain our findings, we hypothesize that GSH may be maintained in hepatocytes after pretreatment with resveratrol, as suggested by the concentration of GSH in liver in our results [41].

Furthermore, it is plausible that improving the redox status by pretreatment with resveratrol may play an important role in enhancing the efflux of arsenic in our study. Conversely, resveratrol has been shown to impair the efflux of cytotoxic drugs in human hepatoma cells, suggesting competitive inhibition of arsenic-transport membrane protein by resveratrol in tumor cells. In addition, combination of resveratrol with As_2_O_3_ may act as a remedial therapy for protecting As_2_O_3_-treated subjects and individuals exposed to arsenicals from the hepatotoxicity of As_2_O_3_ as well as acting as an anticancer agent itself.

In summary, findings from the present and other studies suggest that pharmacological modulators of the redox pathway, such as resveratrol, may be feasible agents for ameliorating As_2_O_3_-induced hepatotoxicity and hepatic dysfunction. Our data support the notion that the protective properties of resveratrol against As_2_O_3_-induced oxidative stress and hepatotoxicity were realized through decreasing the retention of arsenic and improving the redox status of liver tissue. However, further clinical studies are needed to provide data on the clinical use of resveratrol in cases involving arsenic exposure and in an APL patient model.

## Figures and Tables

**Table 1:** The effect of resveratrol on As_2_O_3_-induced changes in serum biochemical parameters.

Treatments	Control	As_2_O_3_	As_2_O_3_ + Rev	Rev
ALT (U/L)	30.75 ± 3.86	83.50 ± 4.65*	40.13 ± 3.84**	33.50 ± 6.25
AST (U/L)	38.75 ± 4.34	82 ± 7.16*	48.75 ± 4.99**	34.50 ± 7.33
Bilirubin (*µ*mol/L)	0.65 ± 0.21	2.03 ± 0.16*	1.10 ± 0.23**	0.61 ± 0.30
Cholesterol (*µ*mol/L)	2.15 ± 0.28	4.15 ± 0.49*	2.35 ± 0.36**	2.04 ± 0.53

Data are mean ± SEM. *P < 0.01 versus control group and **P < 0.05 versus As_2_O_3_-treated group.
